# Uncovering mechanisms of transcriptional regulations by systematic mining of cis regulatory elements with gene expression profiles

**DOI:** 10.1186/1756-0381-1-4

**Published:** 2008-07-17

**Authors:** Qicheng Ma, Gung-Wei Chirn, Joseph D Szustakowski, Adel Bakhtiarova, Penelope A Kosinski, Daniel Kemp, Nanguneri Nirmala

**Affiliations:** 1 Developmental and Molecular Pathways, Novartis Institutes For Biomedical Research Inc, 250 Massachusetts Avenue, Cambridge, MA 02139, USA; 2 Cardiovascular and Metabolism Disease Area, Novartis Institutes For Biomedical Research Inc, 250 Massachusetts Avenue, Cambridge, MA 02139, USA

## Abstract

**Background:**

Contrary to the traditional biology approach, where the expression patterns of a handful of genes are studied at a time, microarray experiments enable biologists to study the expression patterns of many genes simultaneously from gene expression profile data and decipher the underlying hidden biological mechanism from the observed gene expression changes. While the statistical significance of the gene expression data can be deduced by various methods, the biological interpretation of the data presents a challenge.

**Results:**

A method, called CisTransMine, is proposed to help infer the underlying biological mechanisms for the observed gene expression changes in microarray experiments. Specifically, this method will predict potential cis-regulatory elements in promoter regions which could regulate gene expression changes. This approach builds on the MotifADE method published in 2004 and extends it with two modifications: up-regulated genes and down-regulated genes are tested separately and in addition, tests have been implemented to identify combinations of transcription factors that work synergistically. The method has been applied to a genome wide expression dataset intended to study myogenesis in a mouse C2C12 cell differentiation model. The results shown here both confirm the prior biological knowledge and facilitate the discovery of new biological insights.

**Conclusion:**

The results validate that the CisTransMine approach is a robust method to uncover the hidden transcriptional regulatory mechanisms that can facilitate the discovery of mechanisms of transcriptional regulation.

## Background

High-throughput microarray experiments have modernized biological experiments by enabling measurements of expression levels for genes on the genome scale under different conditions. Hundreds or thousands of genes may be differentially expressed between conditions due to the effects of a variety of transcriptional factors or their co-factors. It is challenging to be able to interpret these changes in a biological context. Understanding the transcription regulation mechanisms between transcriptional factors and their target genes is one of the key ways to formulate hypotheses about the root causes of the observed changes.

Unveiling mechanisms of transcription regulation is an active bioinformatics research area. Different approaches have been proposed to discover mechanisms of transcription regulation. Bayesian network approaches have been applied [1] to integrate motif discovery in promoters with the analysis of gene expression data. Some approaches [2] split motifs and gene expression values of regulators to build a decision tree based on the combination of expression ratios of transcription factors and presence/absence of the motifs. Yet other approaches [3,4] fit gene expression data to a linear model using weights depending on whether a transcriptional factor is an inducer or repressor. Mootha *et. al*. [5] uses a two-tailed non-parametric Mann-Whitney (Wilcoxon) rank sum test to determine significance of motifs in promoter regions. The MotifADE method [5] assumes that if up-regulated or down-regulated genes which contain certain transcriptional factor binding sites are co-ordinately regulated, changes in their expression levels could be explained by those transcriptional factors. On the other hand, if genes which contain the same transcriptional factor binding sites are not co-ordinately regulated, there may not be any association between genes and transcriptional factors. In particular, the MotifADE algorithm works in three steps: (1) rank genes based on differential expression between two conditions using the signal-to-noise ratio as the difference metric in descending order (the signal-to-noise ratio is used as opposed to the fold change value based on the expression level since the former also takes into account the standard deviation); (2) For each motif, identify the group of genes whose promoter regions contains the motif; and finally (3) apply the two-tailed non-parametric Mann-Whitney rank sum test to determine if these genes tend to be enriched toward the top or bottom of the ranked list (indicating association) or tend to be randomly distributed on the list (indicating no association).

In our hands, we have observed that two-tailed non-parametric Mann-Whitney rank sum tests used by MotifADE method cannot detect significances of transcriptional factors if they induce the transcription of some genes and repress the transcription of other genes at the same time (see discussion). We have therefore extended the MotifADE method to investigate up-regulated and down-regulated genes separately since a transcriptional factor may simultaneously enhance the transcription of certain genes and inhibit the transcription of other genes. We have also introduced a method to identify the synergistic effects between pairs of transcriptional factors. The CisTransMine method is applied to a mouse C2C12 differentiation dataset [6], where it implicates several known myogenic and cell cycle facts as well as a novel transcriptional factor binding site which regulates known target genes. These results demonstrate that the CisTransMine method is an important tool to discover unknown transcription regulation mechanisms, thus facilitating in extending biological knowledge.

## Results

### Results for known transcriptional factors

**Figure 1:**
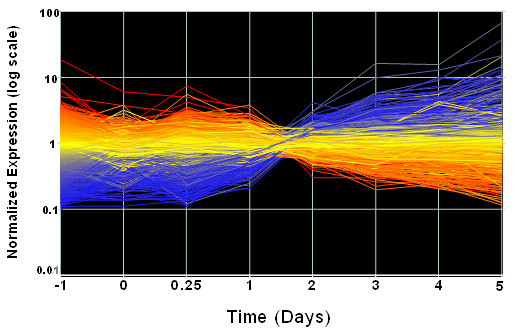
**Gene expression profiles across all time points**. Gene expression profiles across eight time points. It can be observed that major changes occur from Day 1 to Day 2.

We use the mouse C2C12 cell differentiation dataset as a test case [6]. In this experiment, mouse C2C12 myoblast cells were induced to differentiate from myoblasts to myotubes in order to model late stage myogenesis. Cells were cultured in 6-well plates. Induction of differentiation of the C2C12 myoblasts was initiated at Day 0 when cells were confluent by reducing the serum concentration in the wells to 3% v/v. Upon induction of differentiation these mononucleate cells exited the cell cycle and fused to form myotubes. Cells were lysed for RNA preparation. The expression level was measured at eight time-points, with three replicates per time point at days -1, 0, 0.25, 1, 2, 3, 4, 5 post induction. The goal is to identify genes involved in myogenesis. Figure 1 shows gene expression profiles across all time points. It can be observed that the major switch in the expression profiles occurs between Day 1 and Day 2.

**Figure 2:**
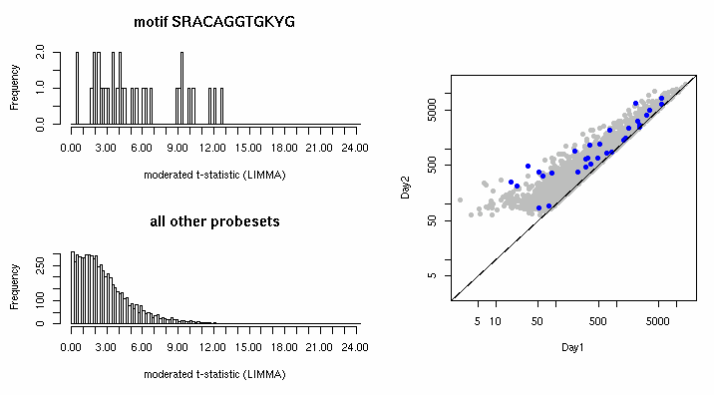
**The distribution of moderated t-values for up-regulated genes containing Myod binding elements in the promoter regions**. The top histogram shows the distribution of moderated t-values for up-regulated MYOD target genes (also depicted as blue dots in the scatter plot), and the bottom histogram shows the distribution of moderated t-values gene expression profiles across all time points for all other up-regulated genes (also depicted as grey dots in the scatter plot).

The CisTransMine algorithm was run on this dataset comparing expression profiles between different time points. Table 1 shows the top 15 transcriptional factors (TF) among up-regulated genes in muscle differentiation between the day 1 and day 2 time points. The top TF among the up-regulated genes is E12, also called E47, which forms heterodimers with MYOD, the second top TF among the up-regulated genes, and is pivotal in controlling muscle transcription [7]. Figure 2 shows the distribution of moderated t-values in up-regulated genes with the MYOD binding elements in their promoter regions. SRF (serum response factor) is required for skeletal muscle growth and maturation [8]. The transcriptional factor C/EBP, which forms heterodimers with C-Jun denoted by CREBP1/CJUN, can activate differentiation-specific genes [9]. MEF2, which is implicated in the muscle contraction process [10], is also enriched since the muscle contraction pathway is up-regulated [6]. Several other top ranked TFs have not been previously linked to muscle and may warrant further investigation into their roles in myogenesis.

**Table 1:** Significant transcriptional factors in up-regulated genes from Day 1 to Day 2

Motif	Occurrence Number	p-value	q-value	Transcription Factors
RRCAGGTGNCV	17	3.87E-05	0.00274	E12
SRACAGGTGKYG	23	0.000125	0.00399	MYOD
RSTGACTNMNW	65	0.000253	0.00399	AP1
GGTACAANNTGTYCTK	34	0.000282	0.00399	GRE
GGACATGCCCGGGCATGTCY	170	0.000306	0.00399	P53
GGGGCGGGGT	245	0.000338	0.00399	SP1
NNRYCACGTGRYNN	38	0.000422	0.00426	USF
CTCTAAAAATAACYCY	11	0.000485	0.00429	MEF2
ATGCCCATATATGGWNNT	67	0.000605	0.00475	SRF
GAAAAGYGAAASY	8	0.00178	0.0126	IRF2
AGATADMAGGGA	15	0.0029	0.018	GATA4
CKSNYTAAAAAWRMCY	4	0.00305	0.018	MMEF2
TGACGTYA	49	0.00389	0.0192	CREBP1/CJUN
RGCAGSTG	14	0.00398	0.0192	MYOGENIN
GGGRATTTCC	75	0.0041	0.0192	NFKAPPAB65

Significant transcriptional factors in up-regulated genes from Day 1 to Day 2. The Motif column shows the consensus binding site sequence for the transcriptional factor. The second column lists the total number genes containing that that transcriptional factor binding sites in the promoter regions. The p-value column illustrates the Mann-Whitney rank sum p-value. The q-value column shows the multiple testing corrected FDR q-value. The transcriptional factor column lists the name of the transcriptional factor which is known to bind to that motif. The total number of up-regulated Refseq genes with raw expression levels at least 100 in Day 2 is 3338.

Table 2 shows a list of statistically significant transcriptional factors in down-regulated genes from Day 1 and Day 2. It has been previously shown that myogenic differentiation in this model is accompanied by cell cycle arrest that is detectable at the transcript level [6]. The results described here implicate a number of TFs that might drive the exit from cell cycle. The transcriptional factors E2F1 and MYC, known regulators of the cell cycle process, are the top enriched transcriptional factors among the down-regulated genes, which implicates E2F1 and MYC as drivers of the previously described cell cycle arrest [6]. The cell cycle checkpoint gene P53 and several known mediators of P53 activity E2F as well as NFY are also among the top enriched TFs [11]. Foxm1, a gene critical for G1/S transition and essential for mitotic progression [12], is also identified by the method. Table 3 illustrates significant synergistic transcriptional factors in down-regulated genes from Day 1 to Day 2. The top interaction pair of transcriptional factors are NFKAPPAB65 and MYC. NFKAPPAB subunits are known to interact with the promoter regions of several genes including MYC (identified here in synergy with NFKAPPAB), Cyclin D1, and SKP2. These interactions are dynamic and depend on the phosphorylation states of NFKAPPAB65 as well as the cell cycle phase [13]. Taken together, these results show that biologically relevant transcription factors involved in muscle differentiation also show statistical significance in the gene expression profiling experiment. Thus one can use CisTransMine to tease out important regulatory processes that are in play under a given perturbation to a system.

**Table 2:** Significant transcriptional factors in down-regulated genes from Day 1 to Day 2

Motif	Occurrence Number	p-value	q-value	Transcription Factors
NKTSSCGC	116	4.47E-11	3.97E-09	E2F1
RACCACGTGCTC	351	2.31E-07	1.03E-05	MYC/MAX
GGGGCGGGGT	253	2.64E-06	7.82E-05	SP1
ARATKGAST	14	6.73E-06	0.000149	FOXM1
TRRCCAATSRN	95	1.56E-05	0.000278	NFY
NNCCACGTGNNN	12	0.000292	0.00415	NMYC
GGACATGCCCGGGCATGTCY	205	0.000327	0.00415	P53
TGACGTYA	65	0.000496	0.00551	CREBP1/CJUN
NBTGGGTGGTCN	12	0.00142	0.014	GLI
NNNNNCCATNTWNNNWN	64	0.00248	0.02	YY1
GCHCDAMCCAG	5	0.00916	0.0592	CP2
TGCTGAGTCAY	5	0.00945	0.0592	NFE2
TCATGTGN	9	0.0124	0.0675	TFE
TGACGTMA	90	0.0136	0.0675	CREB
TWSGCGCGAAAAYKR	10	0.0141	0.0675	E2F

Significant transcriptional factors in down-regulated genes from Day 1 to Day 2. The Motif column shows the consensus binding site sequence for the transcriptional factor. The second column lists the total number of genes containing that motif in the promoter regions. The p-value column illustrates the Mann-Whitney rank sum p-value. The q-value column shows the multiple testing corrected FDR q-value. The last column lists the name of the transcription factor. The total number of down-regulated Refseq genes with raw expression levels at least 100 in Day 1 is 3728.

**Table 3:** Significant synergistic transcriptional factors in down-regulated genes from Day 1 to Day 2

Motif	Occurrence Number	p-value	q-value	Transcription Factors
RACCACGTGCTC_GGGRATTTCC	17	0.00105	0.0122	CMYC NFKAPPAB65
HWAAATCAATAW_TRRCCAATSRN	4	0.0028	0.0122	HNF6 NFY
GCCNNNRGS_ACWTCCK	3	0.00304	0.0122	AP2ALPHA PEA3
AGWACATNWTGTTCT_SGGRNTTTCC	3	0.00523	0.0157	AR CREL
AGACNBCNN_ASMCTTGGGSRGGG	2	0.00859	0.0189	SMAD SP3

Significant synergistic transcriptional factors in down-regulated genes from Day 1 to Day 2. The Motif column shows the consensus binding site sequence for the transcriptional factor where two motifs are separated by an underscore. The second column lists the total occurrence number of genes containing that motif in the promoter regions. The p-value column illustrates the Mann-Whitney rank sum p-value. The q-value column shows the multiple testing corrected FDR q-value. The last column lists the name of the transcription factors. The total number of down-regulated Refseq genes with raw expression levels at least 100 in Day 1 is 3728.

### Results for unknown transcriptional factors

This method was also used to discover novel regulatory elements from this experiment [6]. The elucidation of novel regulatory motifs in the context of a specific cellular function may reveal new pathways and targetable mechanisms related to disease settings. In this paper, the terms "motifs" and "transcriptional factor binding sites" are used interchangeably. Motifs that emerged as potential regulatory elements with statistical significance were screened for functional relevance via luciferase assay. Specifically, motifs were selected in the context of the genes that have a known role in myogenic differentiation and functional pathways that are regulated such as contractility, cell cycle, and mRNA splicing in addition to their statistical significances. The 400 bp DNA sequence surrounding the chosen motifs were analyzed using Transfac for additional transcription factor binding sites, which could potentially influence and complex with the transcription factor identified to bind the unknown novel motif. Table 4 lists the details for tested motifs and other known transcriptional factors within 400 bp DNA sequences surrounding the chosen motifs.

**Table 4:** Tested novel motifs with mutagenesis

Motif	Occurrence number	p-value	Gene symbol	Fold change Ratio	Gene description	Known nearby Transcriptional factor binding sites
gcggaggc	1238	2.57E-06	pck2	0.2	Phosphoenol-pyruvate carboxykinase 2 (mitochondrial)	Oct-1, TFIIA
cgacccgt	95	3.60E-06	myog	5.2	myogenin	SREBP-1, MEF2, MEF3

Novel motifs tested with mutagenesis and their surrounding known transcriptional factor binding sites.

**Figure 3:**
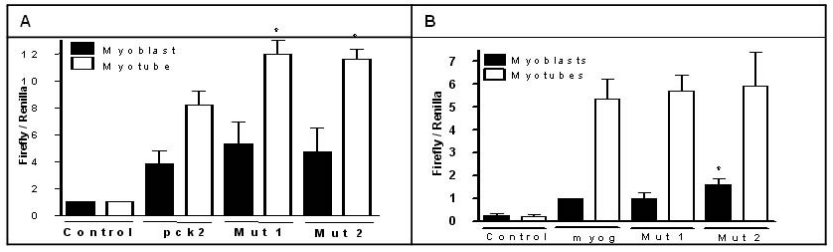
**Luciferase reporter assay results**. Reporter gene assay of pck2 400 bp fragment containing GCGGAGGC motif (A) and myogenin fragment containing CGACCCGT motif (B). There is a change in the reporter activity upon mutagenesis in pck2 constuct and there is no change in myogenin construct. Data normalized to corresponding myoblasts or myotubes transfected with pGL3 promoter vector in the case of pck2 assay (A), Myogenin data was normalized to myoblasts transfected with pGL4.15 containing myogenin construct, because pGL4.15 alone does not have any basal activity. Data represents at least three replicates ± s.e.m. (*, p < 0.05, t-test).

To test for regulatory activity of selected motifs using a reporter gene assay approach, 400 bp sequences were generated by PCR using appropriate primers, and using XhoI restriction sites, these fragments were cloned into the pGL3 promoter reporter vector to assay their transcriptional activity. This relatively large promoter sequence was used due to the potential requirement for contextual surrounding elements for motif function/activity. A 400 bp fragment of the pck2 gene sounding the motif, GCGGAGGC, was cloned from the pck2 promoter into pGL3 promoter firefly luciferase vector and was used to transfect C2C12 myoblasts along with the pGL4.75 Renilla luciferase vector for transfection efficiency. The cells were then split into two plates, cells on one plate were induced to differentiate and the other plate was maintained as undifferentiated myoblasts. Cells transfected with the pGL3 promoter vector without the construct (control), expressed some reporter gene activity, and that reporter activity increased eight fold over the control in the cells transfected with the same vector containing the 400 bp pck2 gene promoter fragment containing the motif, GCGGAGGC (Figure 3A). In order to assess the activity specifically mediated by the motif, the sequence was mutated by random nucleotide substitution, and two different mutant sequences were generated, mutant1 (acgctatc) and mutant2 (ctgcacgc). These mutations led to an increase in the reporter activity beyond that of the wild type motif/promoter, up to twelve fold compared to control. The potential function of this motif, as a negative regulator of gene expression, is consistent with the expression pattern of the pck2 gene within the myogenic program. In contrast, reporter gene activity in C2C12 cells transfected with the pGL4.15 basic vector containing 400 bp of the myogenin promoter with the motif CGACCCGT did not change after mutations were introduced (Figure 3B). Thus, it was deemed that this particular motif has no functional role in the myogenin promoter.

This experiment demonstrated the potential of this method to successfully identify novel functional motifs. Such an approach may be extended to differential gene expression within a variety of disease-related settings and cell types, with potential relevance to disease pathway discovery.

## Discussion

In the post-genomics area, there is a sea of biological data including microarray experimental data. This provides an unprecedented opportunity and challenge to fully decipher the underlying biological system. One aspect of this analysis is to analyze significantly enriched pathways where coordinated but sometimes subtle expression changes are observed among genes [14]. Though the pathway analysis provides a way to see "forests, not individual trees", it can not address the transcription regulation mechanisms which govern the observed gene expression level changes. Thus deciphering transcription regulation mechanisms help characterize the underlying biological process. Different approaches have been proposed to help decipher transcription regulation mechanisms including Bayesian networks, decision trees, and regression models. In this paper, the CisTransMine method has been implemented to identify transcriptional factors involved in biological processes through the analysis of microarray data.

The CisTransMine method not only confirms some known biological knowledge but also reveals potentially novel biological insights. Compared to the results generated by the two-tailed non-parametric Mann-Whitney rank sum test, as used by the MotifADE method shown in Table 5, the CisTransMine method can also identify the transcriptional factors MYOD, AP1, P53, SP1, USF, IRF2, CREBP1/CJUN, and NFKAPPAB65 from up-regulated genes from Day 1 to Day 2 and the transcriptional factors SP1, P53, CREBP1/CJUN, YY1, CP2, NFE2, and TFE from down-regulated genes. Among these transcriptional factors, P53, SP1, and CREBP1/CJUN are significant in both up-regulated genes and down-regulated genes from Day 1 to Day 2 and were missed by the two-tailed non-parametric Mann-Whitney rank sum tests. CisTransMine also identifies additional enriched transcriptional factors which are not supported currently linked to myogenesis (*e.g*. NMYC). CisTransMine did not identify several TFs identified by MotifADE, including HNF4ALPHA and EVI1, and also missed the interaction between E12 and MYOD among up-regulated genes from Day 1 to Day 2. Moreover, only 7066 genes were included in these calculations. As additional transcriptional factors and their target genes are discovered, we will have more coverage on the transcriptional regulation relationships which will result in more comprehensive prediction results.

**Table 5:** Significant transcriptional factors identified by the two-tailed non-parametric

Motif	Occurrence Number	p-value	q-value	Transcription Factors
NKTSSCGC	161	4.28E-12	7.49E-10	E2F1
RACCACGTGCTC	575	3.92E-08	3.43E-06	MYC/MAX
ARATKGAST	15	1.97E-06	0.000115	FOXM1
RGCAGSTG	15	4.79E-06	0.00021	MYOGENIN
VTGAACTTTGMMB	1217	4.24E-05	0.00149	HNF4ALPHA
AGATADMAGGGA	19	0.000158	0.00462	GATA4
ATGCCCATATATGGWNNT	111	0.000203	0.00507	SRF
TWSGCGCGAAAAYKR	10	0.000247	0.00514	E2F
TRRCCAATSRN	159	0.000264	0.00514	NFY
NNCCACGTGNNN	15	0.000495	0.00867	NMYC
CTCTAAAAATAACYCY	14	0.000618	0.00984	MEF2
GGTACAANNTGTYCTK	55	7.00E-04	0.0102	GRE
NBTGGGTGGTCN	15	0.00191	0.023	GLI
RRCAGGTGNCV	27	0.00197	0.023	E12
ACAAGATAA	7	0.00269	0.0288	EVI1

Mann-Whitney rank sum tests from Day 1 to Day 2

Significant transcriptional factors identified by the two-tailed non-parametric Mann-Whitney rank sum tests

from Day 1 to Day 2. The Motif column shows the consensus binding site sequence for the transcriptional factor. The second column lists the total number genes containing that that transcriptional factor binding sites in the promoter regions. The p-value column illustrates the two-tailed Mann-Whitney rank sum p-value. The q-value column shows the multiple testing corrected FDR q-value. The transcriptional factor column lists the name of the transcriptional factor which is known to bind to that motif. The total number of Refseq genes with raw expression levels at least 100 in either Day 1 or Day 2 is 7066.

## Conclusion

In summary, preliminary results identified the relevant transcriptional factors involved in a mouse C2C12 cell model of myogenesis, demonstrating the potential of this method to identify the transcriptional regulatory mechanisms in profiling experiments. We expect that the application of this method to other systems will yield similar results and lead to novel hypotheses regarding the roles of various transcription factors in specific biological systems.

The CisTransMine method was implemented in R, Perl, and C++ and is available upon request. The CisTransMine method was applied to a gene expression profiling experiment of mouse C2C12 skeletal muscle myoblast differentiation to myotubes. The dataset is available from NCBI GEO database [15].

## Methods

### Preparation for promoter sequences

The human, mouse and rat promoter sequences were extracted from the genome assembly as of January 2008. The location of the transcriptional start site was approximated by the first nucleotide in the RefSeq mRNA transcript sequence. For each gene, promoter sequences with respect to their transcripts were extracted according to coordinates of first exons for corresponding transcripts. For each transcript, the region from -2000 bp to +300 bp with respect to the transcriptional start site was extracted. A gene may have several different transcripts, therefore several promoters.

The promoter sequences were masked against repetitive sequences, e.g., LINEs and SINEs with the RepeatMasker program to avoid any Transfac version 11.4 [16] matrix search hits in those repetitive regions. Then orthologous promoter sequences were aligned together with Wconsensus [17]. The orthologous relationships were defined in the NCBI Homologene database as of March 2008. For those promoters with orthologous promoters in human, mouse and rat, a sliding window of 10 nucleotides was used and non-conserved regions were masked out where promoter sequence identities among orthologous promoter sequences had a length of less than 5 nucleotides within a 10 nucleotide window.

### Annotation of promoter sequences

Human-curated transcriptional factor binding sites from the Transfac database were used to record each transcription factor and its regulated genes for human sequences. In addition, the GeneGo Metacore database version 4.6 [18] was used to identify each transcriptional factor and its regulated genes. The Metacore database also reports whether the relationship is the activation or inhibition effect by the transcription regulation, e.g., the human P53 gene regulates 609 target genes by the transcription regulation: among these 609 genes, it transcriptionally activates 206 genes and inhibits 84 genes. Its nature of its interactions with the remaining 319 genes is not explicitly stated. In total, there are a total of 822 human transcriptional factors, 649 mouse transcriptional factors, and 386 rat transcriptional factors in our collection.

### Extraction of unknown transcriptional factor binding sites

Promoter sequence regions which have been annotated as known transcriptional factor binding sites were masked out. The remaining regions contain potentially novel transcriptional factor binding sites. All possible non-degenerative conserved 8-mer and 9-mer motifs which have at least 5 identical nucleotides within a 10 nucleotide window among human, mouse and rat promoter sequences were enumerated. Their true significance would be evaluated in biological experiments.

### Normalization of affymetrix genechip arrays

Affymetrix mouse 430 version 2 microarrays were used to measure gene expression values. Normalization in our analysis was carried out using the GC-RMA normalization method [19]. Values were exponentiated (base 2) to return them to a linear scale and scaled to a 2% trimmed mean of 150. We removed probe sets which have average raw values among replicates less than 100 for both conditions.

### Calculation of the moderated t statistic for each probe set

The traditional student t-test statistic is often used to assess the significance of individual probe sets between two conditions, e.g., treatment group versus control group. However, there are usually only a few replicates (usually three) within each group. Given such a small sample size, it is difficult to estimate the variance reliably. This makes the estimation of the t-statistic problematic. To address this problem, the moderated t-test [20] implemented in the Limma package within the Bioconductor package [21] is adopted to evaluate the significance of individual probe sets between the two groups. The moderated t-test assumes the same distribution for the error variance of all genes in order to estimate the variance of an individual gene with an empirical Bayes method, using posterior residual standard deviations instead of traditional standard deviations, to accommodate for the low number of replicates for each group [20]. Up-regulated genes and down-regulated genes have positive and negative moderated t-values respectively. If a gene is represented by several probe sets, the moderated t-statistic with the highest absolute value is used to represent the moderated t- statistic for that gene.

### Evaluation of the significance of a single motif

**Figure 4:**
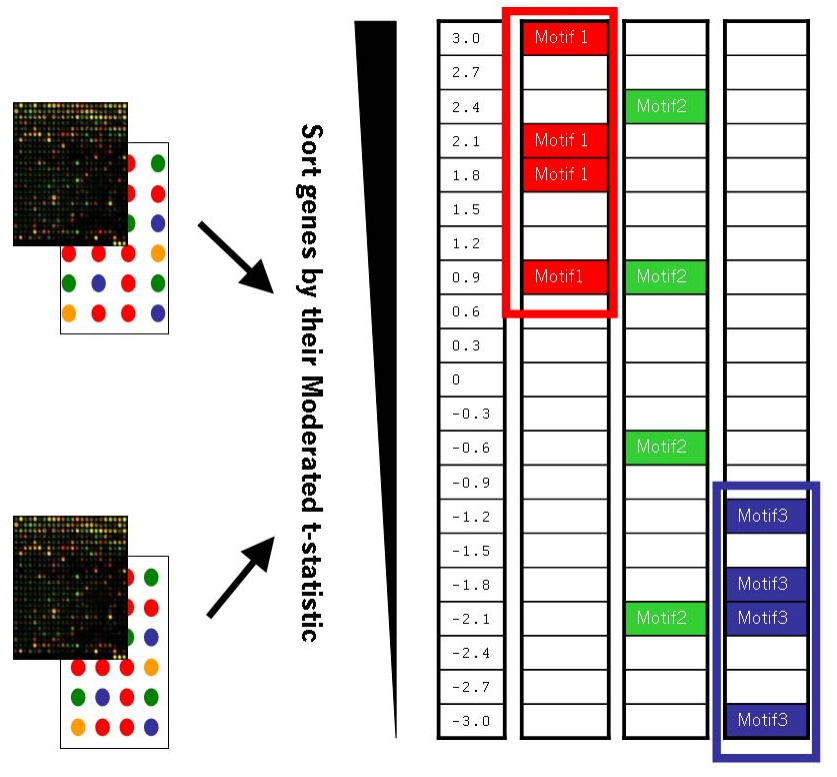
**MotifADE overview**. Overview of MotifADE method: Genes are sorted by their moderated t-test statistic values. Motifs in the promoter regions in these genes are identified. Two-tailed Mann-Whitney rank sum statistics is applied. In this schematic view, Motif 1 is significant in the up-regulated genes. Motif 2 is not significant in either the up-regulated genes or down-regulated genes and Motif 3 is significant in the down-regulated genes.

The CisTransMine method extends the MotifADE framework to identify significant transcriptional factor binding sites enriched between two microarray conditions. MotifADE uses a two-tailed non-parametric Mann-Whitney rank sum U statistic to evaluate the significance of a motif. Specifically, for each motif, t-statistics for all the genes are divided into two groups: one group containing t-statistics for genes having the motif of interest in their promoter region and the other group for genes not having the motif in their promoter regions. The null hypothesis is that there is no difference between the means of the ranks of these two sets of t-statistics; the alternative hypothesis is that the means of the ranks of these two sets are not equal, i.e., genes containing the motif are either up-regulated or down-regulated (Figure 4).

**Figure 5:**
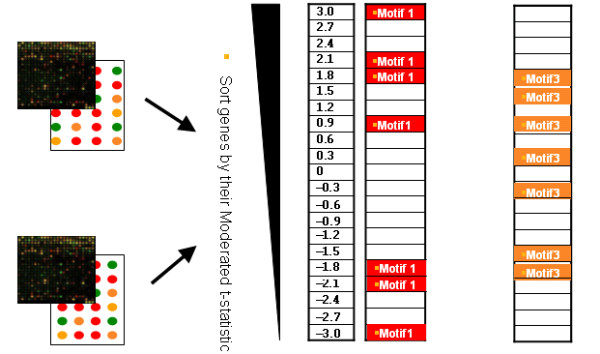
**Problems with the two-tailed Mann-Whitney test**. Motif 1 and Motif 3 would have the same p-values with the two-tailed Mann-Whitney test since only the t-value 0.9 is important and all other t-values from Motif 1 or Motif 3 are symmetric with respect to 0 contributing the same to the rank sum as does t-value 0 even though genes in Motif 1 show higher magnitude changes than genes in Motif 3.

In the case where a transcriptional factor may enhance the transcription of certain genes and repress the transcription of other genes at the same time, the two-tailed Mann-Whitney test might obscure such contexts. Under this situation, a two-tailed Mann-Whitney test cannot detect the significance of that motif since the two-tailed Mann-Whitney test calculates for a given motif, the rank sum for all genes having that motif regardless of up-regulated genes, down-regulated genes, and non-regulated genes. If there are an approximately equal number of up- and down-regulated genes with a particular motif, the statistical significance of the up-regulated genes will be more or less cancelled out by the statistical significance of the down-regulated genes. As a result the motif contained in those genes will be computed to be statistically insignificant. For example, in Figure 5, Motif 1 and Motif 3 would have the same p-values with the two-tailed Mann-Whitney test since only the t-value 0.9 is important and all other t-values from Motif 1 or Motif 3 are symmetric with respect to 0 contributing the same to the rank sum as does t-value 0 even though Motif 1 is more significant than Motif 3, as there are several genes containing motif 1 that are more highly down- or up-regulted relative to the extremes of the genes containing motif 3.

An approach using absolute values was implemented to solve this problem [22] where the absolute enrichment can identify important gene sets that may not be identified by two-tailed methods. The CisTransMine method is proposed to test up-regulated genes and down-regulated genes separately for statistical significance by using the one-tailed non-parametric Mann-Whitney test. For up-regulated (and down-regulated respectively) genes, the null hypothesis is that the mean of the ranks in the up-regulated (and down-regulated respectively) genes containing the motif is equal to the mean of ranks in the up-regulated (and down-regulated respectively) genes not containing the motif; the alternative hypothesis is that the mean of ranks in the up-regulated (and down-regulated respectively) genes containing the motif is greater than (less than respectively) the mean of ranks in the up-regulated (and down-regulated respectively) genes not containing the motif. Thus, significances for motifs in up-regulated genes and down-regulated genes are tested separately.

### Synergistic motifs

In eukaryotic genomes, a synergistic relationship is present when multiple transcriptional factors work in concert to regulate target genes, e.g., combinatorial activities of multiple transcriptional factors regulate the B cell lineage commitment and differentiation [23]. In the CisTransMine method, synergistic relationships between two transcriptional factors are detected in a two-step process. First, the genes containing transcriptional factor A binding sites (TF_A_) and transcriptional factor B binding sites (TF_B_) in the promoter regions can be denoted by TF_A _∩ TF_B_, which is a subset of genes containing both types of binding sites. All the genes containing transcriptional factor A binding sites but not transcriptional factor B binding sites can be denoted by TF_A_- TF_B_. All the genes containing transcriptional factor B binding sites but not transcriptional factor A binding sites can be denoted by TF_B_- TF_A_. For up-regulated (and down-regulated respectively) genes, the necessary conditions for the true synergy between two transcriptional factors to exist are that (1) one-tailed Mann Whitney rank sum test P-value between genes in the set of TF_A _∩ TF_B _and the genes in the set of TF_A_- TF_B _is less than 0.05, (2) one-tailed Mann Whitney rank sum test P-value between genes in the set of TF_A _∩ TF_B _and the genes in the set of TF_B_- TF_A_, is less than 0.05. If the necessary conditions are satisfied, the algorithm proceeds to the second step where the significance of the synergistic relationship between the two transcriptional factors is tested with the same method as that for the single motif with the one-tailed Mann-Whitney rank sum test.

### Multiple testing correction

In order to reduce the false positive rate, multiple testing correction method must be applied to take into account that thousands of null hypotheses are tested at the same time. The multiple testing correction method we adopt is the False Discovery Rate (FDR) q-value [24]. The FDR q-value is a measure of the rate of false discovery from the distribution of p-values. The FDR q-value method is chosen since it can balance between the specificity and the sensitivity without *a priori *p-value cutoff (see reference for details).

## Competing interests

The authors declare that they have no competing interests.

## Authors' contributions

QM carried out the design and implementation of the algorithm and wrote the manuscript. GWC provided the mapping of the Affymetrix probeset to the NCBI refseq sequence. JDS did the quality control of the Affymetrix chips. AB, PAK, and DK did the wet lab work. NRN directed and participated in the project. All authors involved in reviewing and revising the manuscript and approved the final manuscript.
